# Supplementary material for: Rapid and Broad Immune Efficacy of a Recombinant Five-Antigen Vaccine against Staphylococcus aureus Infection in Animal Models
Source: Vaccines (Basel). 2020 Mar 18;8(1):134. doi: 10.3390/vaccines8010134 (PMC7157245; doi:10.3390/vaccines8010134)
Supplement: Supplementary file 1 [file vaccines-08-00134-s001.zip › Supplementary materials and methods 1.docx]

**Supplementary materials and methods 1**

**Cloning, expression and purification of** **recombinant vaccine antigens**

The sequences encoding SpA5, mHI_N2_ and MntC were all from the genome of *S. aureus* MRSA252, and the mSEB sequence was from the the genome of MRSA COL. The DNA sequences encoding recombinant SpA5, mHI_N2_ and MntC were synthesized and inserted into the pGEX-6P-2 vector between the *Not*I and *Bam*HI restriction sites by Sheng Gong Biological Engineering (Shanghai, China). The N-termini of these proteins were fused to a glutathione S-transferase (GST) tag to facilitate protein expression and purification. The DNA sequence encoding recombinant mSEB was synthesized and inserted into the pET22b vector between the *Nco*I and *Bam*HI restriction sites as a tag-less N-terminal construct.

For the expression of recombinant proteins, the SpA5, mHI_N2_ and MntC vectors were transformed into the *E. coli* XL-1 Blue strain (Biovector Science Lab, Beijing, China), and the mSEB vector was transformed into the *E. coli* BL21 (DE3) strain (Biovector Science Lab, Beijing, China). The bacteria were grown in LB medium supplemented with ampicillin (100 μg/mL) until the optical density at 600 nm (OD600) reached 0.6. Isopropyl β-D-1-thiogalactopyranoside (IPTG) was then added to a final concentration of 0.2 mM to induce the expression of the recombinant protein at 16°C overnight.

Different purification protocols were used for each recombinant protein, as briefly described below. The first purification of SpA5, mHI_N2_ and MntC was performed according to the same protocol. In brief, the cells were harvested by centrifugation, and the bacterial pellets were resuspended in PBS and disrupted by ultrasonication. Insoluble cellular material was removed by centrifugation, and the supernatant was mixed with Glutathione Sepharose 4B agarose (GE Healthcare, Piscataway, NJ, USA). After the beads were washed 4 times with PBS, preScission protease (GE) was added to the mixture to cleave the N-terminal GST tag and the mixture was centrifuged. The supernatant containing the GST-free protein was collected for the next purification step.

The supernatant containing the GST-free SpA5 was loaded onto a HiTrap SP HP 5 × 5 mL column (GE Healthcare). The bound protein was eluted with 0 to 1 M NaCl in 20 mM sodium phosphate buffer (pH 7.5). Fractions containing SpA5 were precipitated with ammonium sulfate (20 mM PB (pH 7.5) + 3 M (NH_4_)_2_SO_4,_ 1.4:1.6, V/V) and re-dissolved in His buffer (10 mM His+ 0.9% NaCl, pH 6.0).

The supernatant containing the GST-free mHI_N2_ was loaded onto a HiTrap Capto MMC 5 × 5 mL column (GE Healthcare). The bound protein was eluted with a 0 to 100% gradient of 100 mM NaHCO_3_-NaOH buffer (pH 11.0). Fractions containing mHI_N2_ were precipitated with ammonium sulfate (20 mM PB (pH 7.5) + 3 M (NH_4_)_2_SO_4,_ 1:2, V/V) and re-dissolved in His buffer (10 mM His+ 0.9% NaCl，pH 6.0).

The supernatant containing the GST-free MntC was loaded onto a HiTrap Capto MMC 5 × 5 mL column (GE Healthcare). The bound protein was eluted with 0.1 to 1 M NaCl in 10 mM His buffer (pH 6.0).

For mSEB purification, the cells were harvested by centrifugation, and the bacterial pellets were resuspended in 20 mM PB buffer (pH 5.7) and disrupted by ultrasonication. Insoluble cellular material was removed by centrifugation, and the supernatant was loaded onto a HiTrap SP HP (5 × 5 mL column; GE Healthcare). The bound protein was eluted with a 0 to 0.5 M NaCl in 20 mM PB buffer (pH 6.0). Fractions containing mSEB were then loaded onto a HiTrap Phenyl HP 5 × 5 mL column (GE Healthcare). The bound protein was eluted with 0 to 1 M NaCl in 20 mM PB buffer (pH 6.0).

Then, the solution containing SpA5, mHI_N2_, MntC or mSEB was loaded onto a HiTrap Desalting Sephadex G-25SF 5 × 5 mL column (GE Healthcare) equilibrated in His buffer (10 mM His+ 0.9% NaCl, pH 6.0) for desalination. Fractions containing each protein were loaded onto a HiTrap Q HP 5 × 5 mL column (GE Healthcare) equilibrated with His buffer (10 mM His+ 0.9% NaCl, pH 6.0) to remove endotoxin and then filtered through a 0.22 μm membrane for sterilization.
