# Supplementary material for: Rapid and Broad Immune Efficacy of a Recombinant Five-Antigen Vaccine against Staphylococcus aureus Infection in Animal Models
Source: Vaccines (Basel). 2020 Mar 18;8(1):134. doi: 10.3390/vaccines8010134 (PMC7157245; doi:10.3390/vaccines8010134)

**Supplementary Figures**

**Supplementary Figure S1. The diagram represents timeline for vaccination procedure and S. aureus challenges.**


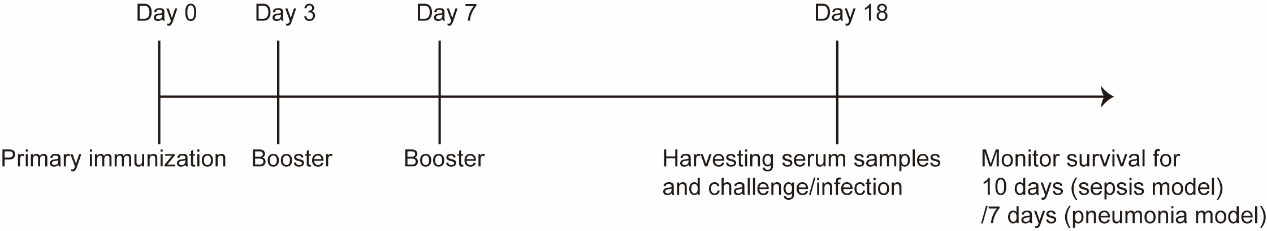


**Supplementary Figure S2. Purity analysis of all 4 proteins (SpA5, mHI_N2_,** **mSEB and** **MntC) by high performance liquid chromatography.**

The chromatographic column used in this method is C3 4.6×150mm, and the samples are analyzed on the machine Agilent 1260. In the test, the column temperature was 60℃, the flow velocity was 0.5mL/min, and the detection wavelength was 280nm. The purity was quantified by area normalization method. (A) The purity of SpA5 was 97.8223%; (B) The purity of mHI_N2_ was 99.2497%; (C) The purity of mSEB was 99.8081%; (D) The purity of MntC was 99.6997%.


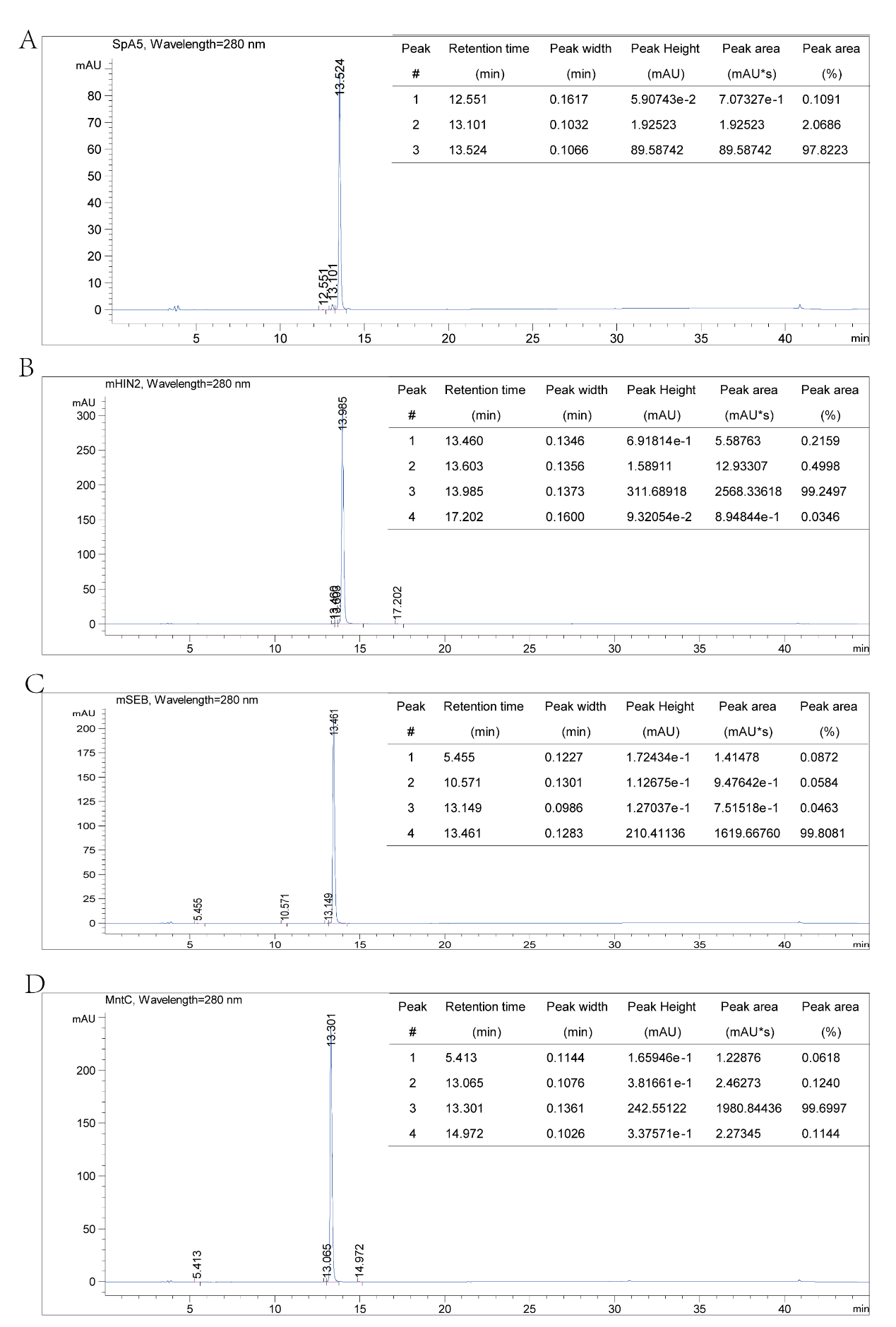


**Supplementary Figure S3. rFSAV-pcAb protected mice from MRSA252 challenge in murine sepsis and pneumonia models.**

(A) Protection of passive immunization with rFSAV-pcAbs in the mouse sepsis model. BALB/c mice (n = 10) were injected intravenously with 100 μl of rFSAV-pcAbs or negative-pcAbs (20 mg/ml). Two hours later, the mice were challenged with MRSA252 at 3.0 × 10^8^ CFUs/mouse by tail intravenous injection. The survival rate was monitored for 10 days. (B) Protection of passive immunization with rFSAV-pcAbs in the mouse pneumonia model. C57BL/6 mice (n = 10) were injected intravenously with 100 μl of rFSAV-pcAbs or negative-pcAbs (20 mg/ml). Two hours later, the mice were challenged with MRSA252 at 5.0 × 10^7^ CFUs/mouse by intratracheal injection. The survival rates were recorded every 12 hours over a 7-day observation period post challenge. (A-B) The Mantel-Cox log-rank test was used to compare differences between passive immunization and control groups.


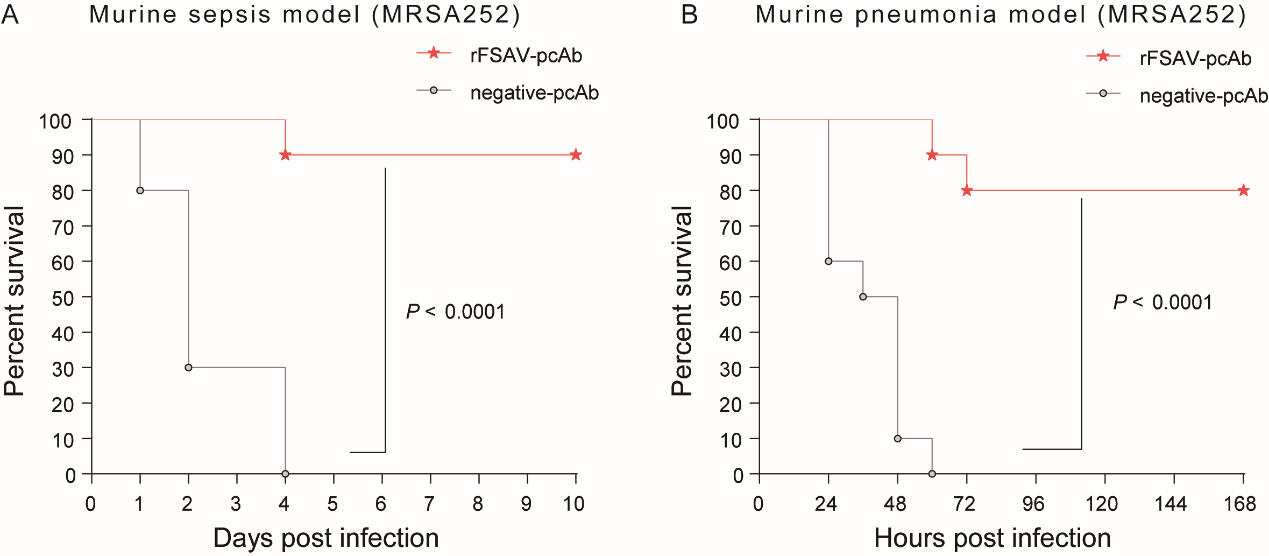

Supplement: Supplementary file 1 [file vaccines-08-00134-s001.zip › Supplementary Figures.docx]
