# Supplementary material for: Rapid and Broad Immune Efficacy of a Recombinant Five-Antigen Vaccine against Staphylococcus aureus Infection in Animal Models
Source: Vaccines (Basel). 2020 Mar 18;8(1):134. doi: 10.3390/vaccines8010134 (PMC7157245; doi:10.3390/vaccines8010134)
Supplement: Supplementary file 1 [file vaccines-08-00134-s001.zip › Supplementary Table 1.doc]

**Supplementary Table 1. The information of *S. aureus* clinical strains used in this study.**

| Strain  name | | Source of strain | Department | Patient's  name | Gender | Age | Source of  sample | Nuc gene^#^ | mecA gene^*^ | Genotyping^&^ |
| --- | --- | --- | --- | --- | --- | --- | --- | --- | --- | --- |
| JN-75 | Jinan military general hospital | | general surgery department | Ji×× | Female | 69 | Blood | + | + | ST239-t437-SCCmecIII |
| CQ-19 | Chongqing southwest hospital | | ICU | Liu× | Female | 22 | Blood | + | + | ST398-t030-SCCmecIII |
| GZ-02 | Guangzhou nanfang hospital | | Traumatic fracture | Zhang× | Male | 21 | Puncture fluid | + | + | ST5-t037-SCCmecIV |
| KM-22 | Kunming medical college | | Burns surgery | Lu×× | Male | 4 | Secretions | + | - | ST121-t030 |
| BJ-04 | Beijing 263 hospital | | ICU | Zhang×× | Male | 69 | sputum | + | + | ST239-t037-SCCmecIII |
| CQ-SA77 | Chongqing xinqiao hospital | | Respiratory department | Zeng× | Male | 19 | sputum | + | + | ST5-t030-SCCmecII |
| GZ-19 | Guangzhou nanfang hospital | | Respiratory department | Lin×× | Male | 54 | sputum | + | + | ST239-t037-SCCmecII |
| SJZ-23 | Shijiazhuang heping hospital | | neurosurgery | Zheng×× | Male | 32 | sputum | + | + | ST59-t570- SCCmecIII |

^#^ nuc gene, which encode the thermonuclease, has been widely used as a specific marker to detect *S. aureus* by PCR-based methods.

^*^ The product of mecA gene is penicillin-binding protein, which resulting in multiple drug resistance.

^&^ Three Genotyping methods were used in this study, including the multiple locus sequence typing (ST), Staphylococal Protein A genetic polymorphism analysis (t) and classification of Staphylococcal cassette chromosome mec (SCCmec).
